# A mixed method feasibility and acceptability study of a flexible intervention based on acceptance and commitment therapy for patients with cancer

**DOI:** 10.3389/fpsyg.2024.1409308

**Published:** 2024-07-03

**Authors:** François Bourgognon, Denise Bechet, Cécile Huin-Schohn, Aurélia Strelow, Laëtitia Demarche, Mireille Guillou, Virginie Adam, Estelle Fall, Abdou Yacoubou Omorou

**Affiliations:** ^1^Department of Supportive Care in Oncology, Institut de Cancérologie de Lorraine, Vandæuvre-lès-Nancy, France; ^2^Departement of Clinical Research, Institut de Cancérologie de Lorraine, Vandæuvre-lès-Nancy, France; ^3^Université de Lorraine, Inserm, INSPIIRE, Nancy, France; ^4^CIC-1433 Clinical Epidemiology, Nancy, France; ^5^The French National Platform Quality of Life and Cancer, Nancy, France

**Keywords:** cancer, MAEva program, mindfulness, acceptance and commitment therapy, quality of life, stress

## Abstract

**Propose:**

This study aimed to propose an innovative, open, and circular program that combines acceptance and commitment therapy (ACT) and mindfulness practices. We assessed its feasibility, acceptability, and first signs of its effect on psychological wellbeing in cancer support treatment.

**Methods:**

A single-center, single-arm, uncontrolled study was performed. Forty adult patients with non-metastatic prostate or breast cancer, newly diagnosed or undergoing treatment (chemotherapy, radiotherapy, hormone therapy), were recruited. Three cycles of three MAEva program sessions (MAEva: Mindfulness meditation, Acceptance, and Commitment to values program) over nine consecutive weeks were proposed. During the total of 12 weeks of follow-up, after attending the first session, patients were free to attend subsequent sessions.

**Results:**

Adherence to the study was high, with participation in an average of 6.8 out of nine sessions. A total of eight patients attended all sessions over the three cycles, and 90% participated in at least one cycle. Furthermore, attendance was associated with a statistically significant improvement in Quality of Life (QoL). Each additional session was associated with a mean increase in overall QoL score of more than one point (β = 1.09 [0.13; 2.04], *p* = 0.02). The fatigue dimensions decreased with session attendance: physical (β = −2.24 [−3.63; −0.85]), emotional (β = −2.60 [−4.11; −1.09]), and interference with daily life (β = −2.33 [−3.95; −0.72]). The qualitative section demonstrated that patients learned skills and shared their ability to “let go”. Patients rated the degree of importance of the program at 8.36/10 (SD ± 1.64).

**Conclusion:**

This study highlights the feasibility and acceptability of an original program that combines ACT and mindfulness practices in cancer patients. Future studies are required to demonstrate the efficacy of the MAEVA program. The MAEva pilot study is registered with ClinicalTrials.gov under the identifier NCT04751201.

**Clinical trial registration:**

https://classic.clinicaltrials.gov/ct2/show/NCT04751201, identifier [NCT04751201].

## Introduction

Cancer is one of the leading causes of morbidity and mortality worldwide, according to the World Health Organization (WHO). Cancer is associated with high levels of distress, particularly anxiety, depression, sleep disturbances, and fatigue (Pitman et al., 2018; Naser et al., 2021). These issues negatively affect patients' Quality of Life (QoL), social relationships, rehabilitation time, medication adherence, and illness behaviors (Artherholt and Fann, 2012; Fann et al., 2012; Grassi, 2020). Cancer patients face significant stressors throughout their care, starting with the wait and announcement of the diagnosis, the therapeutic decision, and the start of treatment. However, clinicians often have few resources to help patients manage stress and improve quality of life (Carlson et al., 2004, 2010; Sellick and Edwardson, 2007). To regulate stress, cancer patients are increasingly turning to complementary medicines and psychological interventions, including mindfulness practices (Huebner et al., 2014; Lyman, 2018; Carlson et al., 2019).

Mindfulness is defined as moment-to-moment awareness with an attitude of non-judgment, acceptance, and openness (Kabat-Zinn, 1990). Mindfulness practices, offered widely in North America and Europe in the form of 8-week programs such as Mindfulness-Based Stress Reduction (MBSR) (Kabat-Zinn, 1990), or Mindfulness-Based Cognitive Therapy (MBCT) (Segal et al., 2002), or Mindfulness-Based Cancer Recovery (MBCR) (Carlson and Speca, 2010), have shown their effectiveness in reducing distress and improving quality of life in cancer patients (Oberoi et al., 2020). For example, a recent meta-analysis of 29 randomized clinical trials of mindfulness-based therapies involving 3,476 cancer patients reported that the interventions reduced anxiety and depression, and improved fatigue, stress, and quality of life (Xunlin et al., 2020). Moreover, in other fields, mindfulness skills have been linked to satisfaction of the three fundamental psychological needs (i.e., autonomy, competence, and relatedness) defined by the self-determination theory (SDT, Ryan and Deci, 2000; Ataşalar and Michou, 2019; Rodríguez-Meirinhos et al., 2021). SDT postulates that satisfaction of one's needs in terms of autonomy (i.e., the feeling of having control of one's life and taking volitional, self-endorsed, and authentic actions), competence (i.e., the feeling of being able to use adaptative learning and having relevant skills to perform chosen actions), and relatedness (i.e., feeling of being included in positive social interactions based on reciprocal feelings of belonging with others) is related to enhanced quality of life and health behaviors (Ryan et al., 2008). In chronic diseases such as HIV, psychological need satisfaction mediated the relationship between mindfulness skills and sleep quality and had an impact on health-related quality of life (Campbell et al., 2019).

Nevertheless, participation in usual mindfulness interventions, such as 8-week programs like MBSR can be difficult, if not impossible, for patients undergoing treatment, because they involve 20–26 h of formal meditation in group sessions of 2.5 h each, plus one full day of practice in silence (6 h), and daily practice of about 45 min at home (Toivonen et al., 2020). Such a high level of requirement is often not compatible with and sometimes contraindicated by the reality of a cancer patient suffering from significant distress or treatment side effects (Creswell, 2017; Taylor et al., 2022).

The proposal of interventions other than the classic 8-week programs, which would be more flexible, open, less formal, and adaptable, is a promising but understudied area (Baminiwatta and Solangaarachchi, 2021; Zhang et al., 2021). Incorporating practices that echo mindfulness, Acceptance and Commitment Therapy (ACT) is a psychotherapy originally developed by Steven C. Hayes and colleagues in the 1980s based on a scientific methodology. It belongs to the Cognitive Behavioral Therapies (CBT) (Hayes et al., 1999). Thanks to various exercises and metaphors, ACT proposes to train mindfulness-related processes without having to use formal learning: acceptance (i.e., willingly accepting the unwanted feelings inevitably elicited by taking difficult actions, particularly those consistent with the patient's hopes, values, and goals), defusion (i.e., stepping back from thoughts that interfere with valued actions and seeing them for what they are), contact with the present moment (i.e., flexibly and purposefully remaining in the present moment by being mindful of thoughts, feelings, bodily sensations, and action potentials, including during distressing experiences), and self-as-context (i.e., keeping balanced and broad perspective on thinking and feeling, such that painful or distressing thoughts and feelings do not automatically trigger maladaptive avoidance behaviors). ACT also offers the opportunity to address two other processes, namely, values (i.e., clarifying fundamental hopes, values, and goals such as being there for one's family, pursuing meaningful work, and so on) and committed action (i.e., cultivating a commitment to doing things in line with identified hopes, values, and goals). Psychological flexibility, defined as the ability to persist in or change behavior when it is in service of valued ends in a particular context, reflects the broader target of the ACT approach (Hayes et al., 2011). The findings of several recent systematic reviews and meta-analyses suggest that ACT is associated with improvements in anxiety, depression, psychological flexibility, and quality of life in patients with cancer (Li et al., 2021; Mathew et al., 2021; Zhang et al., 2023; Jiang et al., 2024). The goal of the present study was to assess the feasibility and acceptability of an original group ACT program with mindfulness practices, to roll the program out on a larger scale. The program called MAEva (M for **M**editation, A for **A**cceptance, and Eva for Commitment to Values) has a very different design from classic mindfulness programs traditionally delivered in 8 weeks. Indeed, the MAEva program is open, circular, and rolling in three sessions, thus enabling a great deal of flexibility in clinical delivery, whereby patients can enter the program at any session (not needing to wait until a group begins), they are invited to participate according to their possibilities, and they can make up for the missed session(s). A proposal such as this would help to address real-world implementation issues in these patients. Therefore, this study tested the feasibility and acceptability of the MAEva program in our center.

## Methods

### Research design

We performed a prospective, single-center, single-arm study testing the feasibility and impact of the MAEva program on patient-reported outcomes among non-metastatic breast and prostate cancer patients. The study was reviewed and approved by the Ethics Committee “Comité de Protection des Personnes Ile de France X” on 15 March 2021 under the number ID-RCB: 2021-A00601-40. All participants provided written informed consent. The study was conducted between September 2021 and July 2022, and all the participants were recruited in our center.

### Participants

A total of 40 patients with non-metastatic breast or prostate cancer were recruited at the Lorraine Institute of Oncology. The inclusion criteria were age 18 years or above, newly diagnosed or undergoing treatment, and able to remain in a seated position for the duration of the sessions (1h30). Although the study was primarily aimed at patients who were in the first phases of treatment (surgery, chemotherapy, radiotherapy), it was also open to patients undergoing hormone therapy. The exclusion criteria were patients currently participating in another meditation program, severe hearing impairment, or severe mental illness.

The program was proposed to patients during consultations by the practitioners (oncologists, nurses, psychologists, etc.). The study was presented to the patients using a brochure. The center's psychiatrist then met with eligible patients, who were given an information leaflet explaining the content of the study. The psychiatrist obtained the patient's written informed consent. Then, the patients were included by the center's psychiatrist who carried out a clinical assessment at the inclusion visit based on a structured interview exploring the main psychiatric disorders [**M**ini **N**europsychiatric **I**nternational **I**nterview (MINI)].

### Procedure

The MAEva program combines ACT and mindfulness practices, and comprises three group sessions of 1 h 30 min each, following a weekly schedule. Each session addresses a specific theme and contains short meditative practices (10–15 min), as well as sharing times with feedback of experience and theoretical contributions: Session 1: Mindfulness Meditation (targeted processes: contact with the present moment and self-as-context), Session 2: Acceptance (targeted processes: acceptance and defusion), and Session 3: Commitment to Values (targeted processes: values and committed action).

**Figure 1:**
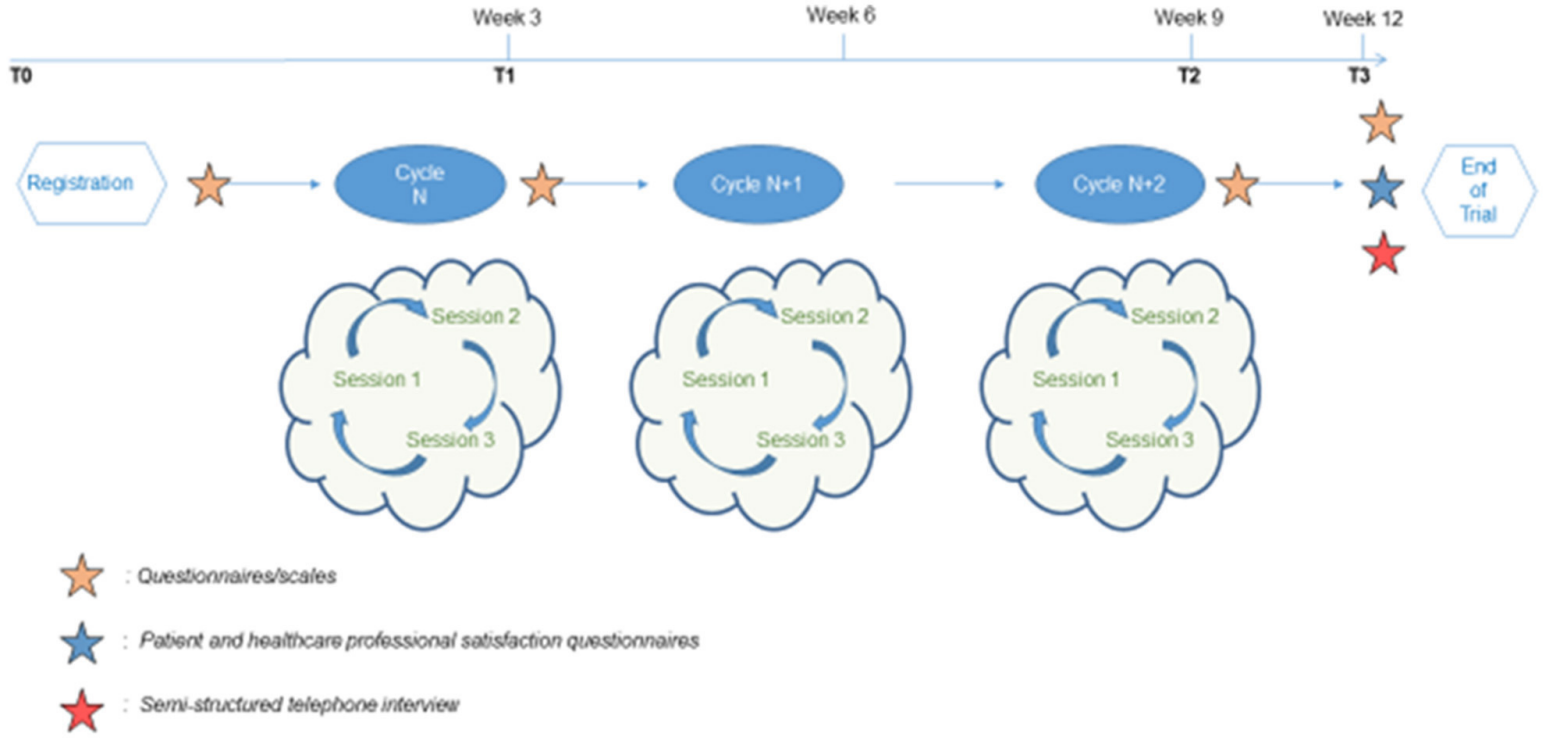
Study process. After enrollment, patient could participate at three cycles of MAEva program within 9 consecutive weeks. Follow-up evaluations are organized in weeks 0, 3, 9, and 12 (or T0, T1, T2, and T3). The MAEva program is an open and circular intervention of three different sessions. Each session addresses a specific theme: Mindfulness meditation, Acceptance and Commitment to values (Session 1, 2 or 3). In this MAEva trial, the maximum duration participation for patient is 12 weeks (graphics program: Microsoft Power Point).

The MAEva program is an open group intervention (i.e., patients can enter the program at any session), and it is circular (i.e., it is possible to do the three sessions in any order and repeat the program to benefit from a training effect). Patients are invited to participate according to their possibilities and without having to commit to carrying out the whole program (i.e., it is fully acceptable for a patient to participate in only one or two of the three sessions of a complete cycle) (Figure 1).

Between sessions, participants were encouraged to engage in daily practice of the ACT therapeutic processes covered. They were also asked to train daily in an original adaptation of the mini-meditation called “three min of breathing space” used in the MBCT program (stop, observe, breathe, expand, and transform reaction into response). Moreover, they were invited to cultivate mindfulness in daily life through informal exercises, that is, to devote oneself attentively to routine activities (e.g., taking a shower, brushing one's teeth, getting dressed, eating, walking, etc.).

The program designer is a psychiatrist, MBCT instructor, ACT therapist, and supervisor.

The facilitators of the sessions were the psychiatrists or psychologists of our center, all trained in ACT, as well as in leading mindfulness sessions, as used in the program. Summary documents were given to the participants at the end of each session, including a summary of the concepts covered during the session and the exercises to practice for the following week. The observation period in this study was 12 weeks for each patient. Moreover, patients could participate in three cycles of the MAEva program over nine consecutive weeks. Follow-up consisted of evaluations scheduled at weeks 0, 3, 9, and 12. In other words, the number of sessions performed per patient could vary from 1 to 9 sessions (one cycle of the MAEva program comprising three sessions; and the cycle repeated three consecutive times).

### Outcome criteria

The main outcome of this study was participation rate, defined as the proportion of patients who participated at least once in each of the three sessions of the program (i.e., attended the Meditation session at least once, the Acceptance session at least once and the Commitment to Values session at least once) over the 9 weeks of the program. Other outcomes of this study were to evaluate the benefit of MAEva on wellbeing in terms of QoL, fatigue, anxiety and depression, process and mechanism, as well as adherence to the program.

## Measures

### Quantitative data

Evaluations were performed at inclusion (T0), week 3 (T1), week 9 (T2), and week 12 (T3), and data were recorded using Cleanweb software (Figure 1). Follow-up at T1 and T2 corresponded to the intervention period, while T3 corresponded to the follow-up evaluation 3 weeks post-intervention. At each follow-up period, an e-mail was automatically sent to patients, inviting them to complete the online questionnaire. Participants who did not have internet access or did not have an e-mail address had the option of completing the questionnaires in paper format.

Sociodemographic (age, sex, marital status, education level, professional situation) and clinical data (body mass index, WHO performance status, localization of cancer, treatment) were collected at enrollment.

Patient-reported outcome measures (PROMs) were assessed using three questionnaires. The Global Health and Quality of life (QoL) scale in cancer patients was assessed using the validated French version of the EORTC QLQC-30 (European Organization for Research and Treatment of Cancer 30-item QoL questionnaire) (Aaronson et al., 1993). It is a cancer-specific QoL self-administered questionnaire with 30 items grouped into five functional dimensions (PF: physical, RF: role, CF: cognitive, EF: emotional, and SF: social), nine symptomatic dimensions (fatigue, pain, nausea and vomiting, dyspnea, sleep disturbance, loss of appetite, constipation, diarrhea, and financial impact), and one global health status (GHS) dimension (example items from the questionnaire: *During the past week, have you had pain? Were you limited in doing either your work or other daily activities? Have you had difficulty remembering things? Did you feel irritable? Has your physical condition or medical treatment interfered with your social activities?*). The score of each dimension was calculated and normalized on a scale from 0 to 100 (high QoL). Cronbach's alpha value ranged from 0.70 for pain, to 0.88 for fatigue, and to 0.80 for GHS.

Cancer-related fatigue was evaluated using the QLQ-FA12 questionnaire (Weis et al., 2017)—a multidimensional instrument consisting of 12 items—to be used in conjunction with the basic quality-of-life questionnaire (QLQ-C30). This module assesses the physical, cognitive, and emotional aspects of cancer-related fatigue (example items from the questionnaire: *During the past week, did you feel sleepy during the day? Did you have trouble thinking clearly? Did you feel discouraged?*). Cronbach's alpha value ranged from 0.88 for emotional fatigue to 0.93 for cognitive fatigue.

Anxiety and depression were measured using the 14-item Hospital Anxiety and Depression Scale (HADS) (Zigmond and Snaith, 1983). This scale is frequently used to assess psychosocial outcomes in cancer patients, and its reliability and validity have been confirmed (Zhang et al., 2015; example items from the questionnaire: *I feel tense or ‘wound up', I can laugh and see the funny side of things, I get sudden feelings of panic*). Each subscale is scored from 0 to 21, with higher scores indicating greater anxiety and depression; 0–7 is generally considered within a normal range. Cronbach's alpha ranged from 0.63 for anxiety to 0.82 for depression.

The number of sessions attended and the topics covered were assessed to calculate participation data. An end-of-study satisfaction questionnaire was given to participants at T3 (week 12) to collect their opinions and feedback about the conduct of the MAEva program. In particular, they were asked to rate the degree of importance that the program had for them on a scale ranging from 0 (not important at all) and 10 (very important), and to explain why. Several other open questions were also included to evaluate the benefits and obstacles of the program. A satisfaction questionnaire was also given to the facilitators of the sessions.

### Qualitative data

Qualitative data were collected to evaluate the processes and mechanisms of action involved during the MAEva program using semi-structured telephone interviews conducted at T3 (week 12) with the first 10 participants (Figure 1). An interview guide was developed in advance to specify the various questions to be addressed. The interviews were recorded and then transcribed for qualitative framework analysis.

## Data analyses

### Sample size and the smallest detectable difference

The inclusion period was 7 months corresponding to a potential inclusion of 40 patients. Thus, the smallest detectable difference (SDD) was calculated with this sample size. Change in anxiety score was used to define the smallest detectable difference (As the program was expected to have the greatest impact on this outcome). The SDD for anxiety was calculated with a 5% type I error and 80% power, assuming a normal distribution of the 12-week change and a standard deviation (SD) of 4.0. In these conditions, this study would be able to detect a minimal change in anxiety score from before to after the intervention of 1.82 points. Knowing that a difference of 1.7 points in the anxiety score was considered the minimum threshold beyond which the difference was clinically relevant (Lemay et al., 2019); this estimated SDD seemed acceptable to us.

### Quantitative data

Baseline sociodemographic and clinical characteristics of the study sample were assessed using descriptive statistics (mean ± SD; number (N), and percentage). The scores were calculated for the different dimensions of the PROMs and their change at each follow-up time (weeks 3, 9, and 12) compared to inclusion.

For the main objective of this study, the level of patient participation was assessed for the various sessions offered (total number of sessions, total number of mindfulness meditation sessions, total number of acceptance sessions, and total number of value commitment sessions). Feasibility of the study was defined as participation at least once in each of the three sessions of the program over the 9-week period.

Finally, the association between the level of participation in the sessions and changes in PROMs was analyzed using linear mixed models for repeated measures with the patient as a random effect. The mixed models were first univariate and then multivariate, and they were adjusted for cancer type, WHO status, prior surgery, and receipt of complementary treatment.

### Qualitative data

Qualitative data from the semi-structured interviews were analyzed according to the framework analysis approach (Gale et al., 2013). For each interview, the transcript was read several times to become familiar with the discourse of the interviewee. With each interview transcript, informative statements were extracted into units of meaning and categorized into different themes. The 10 interviews were initially categorized into themes corresponding to the 6 ACT processes involved in the intervention (i.e., contact with the present moment, self-as-context, acceptance, defusion, values, and committed action). Then, to include other themes that participants expressed, the coding was expanded using a second codebook based on self-determination theory to identify units of meaning relevant to the three main psychological needs (i.e., autonomy, competence, and relatedness).

## Results

### Study sample characteristics

All participants were included in the statistical analysis using the intention to treat (ITT) analysis.

Table 1 shows the baseline sociodemographic, clinical, and PROMs data. The study sample was predominantly female (37/40, 92.5%) with an average age of 50.4 years [min = 31 and max = 70]. Approximately, 60% were married, over 90% had a high school diploma or higher education, and 47.5% were intermediate professionals. Clinically, there were 37 cases of breast cancer and three cases of prostate cancer.

**Table 1:** Baseline sociodemographic, clinical and perceived health of the study sample.

	***N***	**%/Mean**	SD^*^
Age (years)	40	50.4	10.1
**Sex**			
Female	37	92.5	
Body mass index (kg/m^2^)	40	24.2	4.6
**WHO performance status**			
0	6	15.0	
1	10	25.0	
2	24	60.0	
**Localization of cancer**			
Prostate	3	7.5	
Breast	37	92.5	
**Ongoing treatment**			
Yes	34	85.0	
**Treatment**			
Missing	6		
Chemotherapy	17	50.0	
Radiotherapy	12	35.3	
Targeted therapy	1	2.9	
Hormone therapy	4	11.8	
**Surgery**			
Yes	25	62.5	
**Complementary treatment**			
Yes	28	70.0	
**Marital status**			
Single	5	12.5	
Married	24	60.0	
Living martially	9	22.5	
Widowed/separated	2	5.0	
**Education level**			
<High school	2	5.0	
High school diploma	2	5.0	
University or higher	36	90.0	
**Professional situation**			
Not working	29	72.5	
Working	11	27.5	
**Occupational categories**			
Managerial staff, higher intellectual professions	12	30.0	
Intermediate professions	19	47.5	
Employees	2	5.0	
Retired	6	15.0	
Other	1	2.5	
**Having caregiver**			
Yes	2	5.0	
**Quality of life (QLQ-C30)**			
Physical functioning	40	82.5	15.9
Mental functioning	40	73.3	24.1
Cognitive functioning	40	71.7	29.5
Emotional function	40	56.9	26.3
Social functioning	40	73.8	29.5
Fatigue	40	42.8	25.2
Pain	40	24.6	21.7
Nausea and vomiting	40	7.9	13.1
Insomnia	39	53.8	31.2
Constipation	40	25.8	35.8
Dyspnea	40	25.0	30.0
Diarrhea	40	14.2	21.2
Loss of appetite	39	10.3	20.5
Financial difficulties	39	7.7	16.2
Global Health status	40	62.1	17.0
**Anxiety and depression (HADS)**			
Anxiety (0–100)	37	50.8	10.5
Depression (0–100)	40	41.7	7.9
Total (0–100)	37	46.5	6.5
**Depression classes**			
No depression	10	25.0	
Suspected depressive symptoms	25	62.5	
Definite depressive symptoms	5	12.5	
**Anxiety classes**			
No anxiety	1	2.7	
Suspected anxiety symptoms	16	43.2	
Definite anxiety symptoms	20	54.1	
**Cancer-related fatigue (FA-12)**			
Physical fatigue	40	42.3	26.6
Emotional fatigue	40	42.2	31.7
Cognitive fatigue	40	28.3	33.2
Interference with daily life	40	34.2	28.7
Social sequelae	40	10.8	24.3

^*^Standard deviation; N, Number of participants; QLQ, Quality of Life Questionnaire; HADS, Hospital Anxiety and Depression Scale; QLQ-FA12: and QoL Module Measuring Cancer-Related Fatigue.

None of the patients included in the study had adhered to a meditation program before inclusion, none were currently on another meditation program (exclusion criteria), and none engaged in regular meditation practice at the time of their inclusion.

Of the participants, 62.5% had undergone surgery and 85% were currently undergoing treatment.

At inclusion, patients had relatively high QoL scores, especially for the physical, psychological, social, and cognitive dimensions. The symptom dimensions of the QLQ-C30 were relatively low. According to the HADS questionnaire scores, more than three-quarters of patients probably (62.5%) or definitely (12.5%) had symptoms of depression, and almost all of the patients (97.8%) probably (43.2%) or definitely (54.1%) had symptoms of anxiety. There was an intermediate level of fatigue measured by the FA-12 questionnaire at inclusion, particularly for physical and emotional fatigue.

### Main outcome

Overall, attendance in the MAEva program was excellent, with patients attending an average of 6.8 out of 9 sessions (min = 2, max = 9) (Table 2). A total of 8 of 40 participants (20%) attended all the 9 sessions. The level of participation in the thematic sessions was similar, with 23 (57.5%) attending all the Mindfulness meditation sessions, 18 (45%) attending all the Acceptance sessions, and 17 (42.5%) attending all the Commitment to Values sessions. The feasibility was excellent, with almost 90% of patients attending at least one session of each type.

**Table 2:** Participation in the MAEva program.

	***N***	**Mean (%)**	SD^*^	**Median**	**Q1**	**Q3**	**Min**	**Max**
Total number of sessions	40	6.8	2.1	7.0	6.0	8.0	2.0	9.0
**Overall level of participation in the program**								
At least one session on each theme	5	12.5						
2–3 sessions on each theme	27	67.5						
All sessions	8	20.0						
Number of Meditation sessions	40	2.4	0.9	3.0	2.0	3.0	0.0	3.0
**Complete participation in meditation sessions**								
No	17	42.5						
Yes	23	57.5						
Number of Acceptation sessions	40	2.3	0.8	2.0	2.0	3.0	1.0	3.0
**Complete participation to acceptation sessions**								
No	22	55.0						
Yes	18	45.0						
Number of commitment to values sessions	40	2.2	0.8	2.0	1.5	3.0	1.0	3.0
**Complete participation in commitment to values sessions**								
No	23	57.5						
Yes	17	42.5						
Number of first cycle sessions	40	2.6	0.5	3.0	2.0	3.0	2.0	3.0
Number of second cycle sessions	40	2.0	1.0	2.0	1.0	3.0	0.0	3.0
Number of third cycle sessions	40	2.1	1.1	3.0	2.0	3.0	0.0	3.0
**Complete participation in the first cycle sessions**								
No	15	37.5						
Yes	25	62.5						
**Complete participation in the second cycle sessions**								
No	23	57.5						
Yes	17	42.5						
**Complete participation in the third cycle sessions**								
No	19	47.5						
Yes	21	52.5						
**Attendance at each session**								
**Session 1**								
Yes	40	100.0						
**Session 2**								
No	7	17.5						
Yes	33	82.5						
**Session 3**								
No	8	20.0						
Yes	32	80.0						
**Session 4**								
No	11	27.5						
Yes	29	72.5						
**Session 5**								
No	14	35.0						
Yes	26	65.0						
**Session 6**								
No	13	32.5						
Yes	27	67.5						
**Session 7**								
No	14	35.0						
Yes	26	65.0						
**Session 8**								
No	9	22.5						
Yes	31	77.5						
**Session 9**								
No	12	30.0						
Yes	28	70.0						

^*^Standard deviation; N, Number of participants.

### Secondary outcomes

Adherence to the program was associated with a statistically significant improvement in QoL, particularly in its functional dimensions (Tables 3, 4). For example, participation in each additional session was associated with a mean increase in overall QoL score of more than one point at each measurement time (β = 1.09 [0.13; 2.04], *p* = 0.02). The psychological and emotional dimensions were those that were most positively impacted by the sessions with, respectively, β = 2.27 [0.84; 3.71] and β = 2.31 [1.12; 3.51]. Participation level was also associated with a significant improvement in fatigue. The fatigue dimensions decreased with session attendance: physical (β = −2.24 [−3.63; −0.85]), emotional (β = −2.60 [−4.11; −1.09]), and interference with daily life (β = −2.33 [−3.95; −0.72]). Conversely, the nausea and vomiting dimension showed a negative association with presence at the sessions (β = 1.11 [0.45; 1.78]). No statistically significant association was found between the number of sessions and changes in anxiety and depression scores. Finally, on the satisfaction questionnaires, patients reported an average score of the degree of importance that the program had for them of 8.36/10 (SD ± 1.64).

**Table 3:** Description of changes in quality of life, fatigue, anxiety, and depression during follow-up.

	**Inclusion**			**Week 3**			**Week 6**			**Week 12**			
	***N***	**Mean**	**SD** ^*^	***N***	***M*** **ean**	**SD** ^*^	***N***	**Mean**	**SD** ^*^	***N***	**Mean**	**SD** ^*^	*p* ^**^
Physical functioning	40	82.5	15.9	33	81.4	19.5	36	86.1	17.3	33	83.4	18.1	0.71
Mental functioning	40	73.3	24.1	33	73.2	30.0	36	78.7	24.8	33	83.3	24.3	0.30
Cognitive functioning	40	71.7	29.5	32	69.8	32.1	36	79.2	22.7	33	76.8	28.2	0.48
Emotional functioning	**40**	**56.9**	**26.3**	**32**	**66.1**	**21.4**	**36**	**73.6**	**17.9**	**33**	**73.0**	**22.0**	**0.004**
Social functioning	40	73.8	29.5	32	79.2	22.4	36	82.9	23.1	33	77.3	29.7	0.50
Fatigue	40	42.8	25.2	33	45.1	26.6	36	36.7	19.3	33	36.5	23.9	0.33
Pain	40	24.6	21.7	33	29.8	28.2	36	16.2	17.1	33	23.2	29.4	0.14
Nausea and vomiting	**40**	**7.9**	**13.1**	**33**	**9.1**	**15.1**	**36**	**3.2**	**6.7**	**33**	**3.0**	**7.7**	**0.04**
Insomnia	**39**	**53.8**	**31.2**	**33**	**31.3**	**26.3**	**36**	**37.0**	**32.6**	**33**	**33.3**	**30.0**	**0.006**
Constipation	40	25.8	35.8	33	19.2	31.2	36	9.3	15.1	33	19.2	27.7	0.09
Dyspnea	40	25.0	30.0	32	22.9	28.6	36	18.5	20.2	33	21.2	24.7	0.74
Diarrhea	40	14.2	21.2	32	12.5	18.5	36	11.1	19.5	33	9.1	22.5	0.75
Loss of appetite	39	10.3	20.5	32	11.5	21.8	35	5.7	12.7	32	7.3	20.3	0.58
Financial difficulties	39	7.7	16.2	32	9.4	19.4	35	8.6	21.9	33	11.1	19.8	0.89
Global Health status	40	62.1	17.0	31	62.4	18.2	36	69.4	16.2	33	69.9	20.0	0.10
Anxiety (0–100)	37	50.8	10.5	32	48.5	12.7	36	48.9	10.5	31	49.6	9.6	0.81
Depression (0–100)	40	41.7	7.9	33	41.7	10.1	36	42.5	7.9	34	39.5	7.6	0.49
Total (0–100)	37	46.5	6.5	32	45.1	7.0	36	45.7	5.4	31	44.3	5.9	0.54
Physical fatigue	40	42.3	26.6	32	42.5	24.6	35	29.3	21.2	33	33.9	27.9	0.07
Emotional fatigue	**40**	**42.2**	**31.7**	**32**	**29.9**	**27.7**	**35**	**24.1**	**23.9**	**33**	**23.9**	**29.1**	**0.01**
Cognitive fatigue	**40**	**28.3**	**33.2**	**32**	**18.7**	**29.9**	**35**	**8.1**	**16.4**	**33**	**19.7**	**29.3**	**0.02**
Interference with daily life	40	34.2	28.7	32	33.3	29.3	35	21.0	19.9	33	27.3	28.2	0.13
Social sequelae	40	10.8	24.3	32	13.5	27.9	35	8.6	20.4	33	15.2	30.2	0.72

^*^Standard deviation.

^**^Chi-square test for qualitative variables, test stemming from an analysis of variance for quantitative variables. Bold indicates statistically significant (*p*-value <0.05).

**Table 4:** Association between level of participation and change in patient-reported outcomes measures.

	**Univariate β [95% CI]**	***P*-value**	Multivariate^*^β [95% CI]	***P*-value**
**Quality of life (QLQ-C30)**				
Global health status	**1.20 [0.24; 2.15]**	**0.01**	**1.09 [0.13; 2.04]**	**0.02**
**Functional dimensions of QLQ-C30**				
Physical functioning	**1.48 [0.70; 2.27]**	**0.0003**	**1.42 [0.64; 2.21]**	**0.0005**
Mental functioning	**2.45 [1.02; 3.88]**	**0.0009**	**2.27 [0.84; 3.71]**	**0.002**
Cognitive functioning	0.81 [−0.22; 1.86]	0.12	0.78 [−0.26; 1.83]	0.14
Emotional function	**2.44 [1.26; 3.63]**	**<0.0001**	**2.31 [1.12; 3.51]**	**0.0002**
Social functioning	**2.24 [0.62; 3.85]**	**0.007**	**2.05 [0.42; 3.68]**	**0.01**
**Symptomatic dimensions of QLQ-C30**				
Fatigue	**-1.76 [**–**2.87;** –**0.66]**	**0.002**	–**1.65 [**–**2.75;** **−0.54]**	**0.003**
Pain	–**1.94 [**–**3.34;** –**0.53]**	**0.007**	–**1.92 [**–**3.33;** **−0.51]**	**0.008**
Nausea et vomiting	**1.04 [0.38;** –**1.70]**	**0.002**	**1.11 [0.45; 1.78]**	**0.001**
Insomnia	−0.87 [−2.58; 0.83]	0.31	−0.61 [−2.32; 1.08]	0.47
Constipation	−1.40 [−2.91; 0.09]	0.06	−1.44 [−2.95; 0.06]	0.06
Dyspnea	−0.86 [−2.14; 0.42]	0.18	−0.81 [−2.10; 0.46]	0.21
Diarrhea	−1.03 [−2.38; 0.30]	0.12	−0.92 [−2.27; 0.43]	0.18
Loss of appetite	0.26 [−0.82; 1.34]	0.63	0.35 [−0.73; 1.43]	0.52
Financial difficulties	−0.21 [−1.14; 0.71]	0.65	−0.18 [−1.12; 0.74]	0.69
**Anxiety-depression (HADS)**				
Overall score	0.06 [−0.31; 0.44]	0.73	0.04 [−0.33; 0.42]	0.81
Anxiety score	−0.15 [−0.74; 0.42]	0.59	−0.19 [−0.77; 0.39]	0.51
Depression score	0.27 [−0.14; 0.70]	0.19	0.29 [−0.12; 0.72]	0.16
**Fatigue (QLQ-FA12)**				
Physical fatigue	–**2.41 [**–**3.80;-1.02]**	**0.0008**	–**2.24 [**–**3.63;** **−0.85]**	**0.001**
Emotional fatigue	–**2.73 [**–**4.24;−1.23]**	**0.0005**	–**2.60 [**–**4.11;** **−1.09]**	**0.0009**
Cognitive fatigue	−1.23 [−2.55; 0.08]	0.06	−1.12 [−2.44; 0.19]	0.09
Interference with daily life	–**2.55 [**–**4.16;** **−0.95]**	**0.002**	–**2.33 [**–**3.95;** **−0.72]**	**0.005**
Social sequelae	−0.10 [−0.98; 0.77]	0.81	−0.088 [−0.97; 0.79]	0.84

^*^Multivariate analysis adjusted for WHO performance status, localization of cancer, surgery, and ongoing treatment (β: regression coefficient; 95% CI: 95% Confidence Interval; and significant p-value <0.05); QLQ, Quality-of-Life Questionnaire; HADS, Hospital Anxiety and Depression Scale; QLQ-FA12: and QoL Module Measuring Cancer-Related Fatigue. Bold indicates statistically significant (*p*-value <0.05).

### Post-intervention interview outcomes

Table 5 presents the number of units of meaning relevant to the ACT process. Using the first codebook based on ACT processes, the following themes were extracted: (1) committed action, (2) contact with the present moment, (3) self-as-context, (4) defusion, (5) values, and (6) acceptance. They are presented from the most to the least commonly expressed.

**Table 5:** Number of units of meaning relevant to the ACT process.

**Processes**	**Numbers of units of meaning**	**Presentation of some translated extracts from semi-structured interviews**
Committed action	24	‘It has given me more in the way of decision-making. I am no longer trying to put everything into a 24-h day.”.....“It's easier for me to sort out my commitments' (woman; 49 years old)
Contact with the present moment	14	“Gave myself a few moments Take a break Take stock of the current situation” (woman; 49 years old)
Self-as-context:	13	“The importance of refocusing on yourself and not letting your little *monkey* mind invade you all the time” (woman; 49 years old)
Defusion :	11	“Learn to let your thoughts flow and not to loop around them” (woman; 46 years old)
Values	8	“I find it easier to distinguish between what is important and what is not, for me in any case” (woman; 34 years old)
Acceptance	5	“and I'm able to accept my emotions, feel them, accept them, and get along with them, which I wasn't able to do until now” (woman; 51 years old)

Through their attendance at sessions, application of daily exercises, involvement in long-term practice, and transmission of what they have learned to members of their entourage, participants demonstrated their commitment to applying mindfulness during and after the MAEva program *(Committed action)*. The interview analysis also reveals a strong appropriation of the “being present” process. Thanks to the MAEva program, participants developed some capacities, such as anchoring themselves in the present moment, identifying mind wandering, and focusing on bodily sensations, enabling them to experience the present moment more fully *(Contact with the present moment)*.

Through the participants' discourse, it was also possible to perceive the development of the ability to “let go” during the program, giving them access to another way of experiencing the present moment, more in line with their authentic selves, and by the “self-as-context” perspective *(Self-as-context)*. The regular use of an original adaptation of the mini-meditation called “*three minutes of breathing space”* used in the MBCT program was reported by the majority of the participants after the program. This underlines the development of the defusion process, which refers to the ability to distance oneself from thoughts and observe them without judgment or an impulse to react *(Defusion)*.

Finally, most participants were receptive to the values process during the sessions of MAEva program, as seen in their words relating their ability to give great meaning to their lives and to identify their sources of reinforcement *(Values)*. Although less represented in participant discourse, the acceptance process was illustrated through recurrent expressions such as “welcome, deal with, not fight against” thoughts and emotions *(Acceptance)* (Table 5).

Using the second codebook based on basic psychological needs, three themes were extracted: (1) competence need; (2) autonomy need; and (3) relatedness need (Table 6).

**Table 6:** Number of units of meaning relevant to three basic psychological needs of self-determination theory.

**Processes**	**Numbers of units of meaning**	**Presentation of some translated extracts from semi-structured interviews**
Competence need	36	“Better management of difficulties. Learning to let go of certain difficulties This gave me great serenity about my illness, Distance from the situation” (woman; 46 years old)
Autonomy need	19	“You have given us the right tools. Now it's up to us to use them” (man; 68 years old)
Relatedness need	11	“To be surrounded by other people who have been through the same ordeal, the same journey as me. Mutual understanding” (woman; 51 years old)

The *Competence* need is illustrated in the participant discourse highlighting the ability to autonomously apply the techniques taught in the program. The MAEva program enabled patients to better manage the anxiety and/or pain associated with cancer through their ability to apply the meditative practices, observe their inner processes, learn self-awareness, and thanks to emotions and disease management. The *Autonomy* need is perceptible in the patients' discourse through their decision to participate in the group sessions and to choose whether to use the techniques learned during the MAEva program. Moreover, this need is also obvious in the way they organize their practice and decide to get involved in other activities. Finally, the need for *relatedness* is also reinforced, with the importance of belonging to a group, as expressed by more than half the participants (Table 6).

## Discussion

This study highlights the feasibility and acceptability of an original program using ACT with mindfulness practices for cancer patients. To obtain a homogeneous population sample, patients with non-metastatic breast or prostate cancer were either recruited, newly diagnosed, or undergoing treatment. Our center is particularly involved in the treatment of breast cancer. Most patients included in this study were women. Conducting this study confirmed to us that the inclusion of women is easier in our center for this type of intervention. As this is a feasibility study, we did not wish to exclude the three patients with prostate cancer from our analyses. Indeed, in our view, the MAEva program seems sufficiently flexible to be offered to a majority of cancer patients, regardless of the location or stage of the disease, which could be the subject of future studies.

The program attendance throughout the observation period was high, and almost all patients received the full intervention (which was defined as participation at least once in each of the three types of sessions of the MAEva program). In line with these results, the MAEva program was appreciated by the patients, and they reported that it had a high degree of importance for them.

Our results indicate that participation in the MAEva program was associated with an improvement in QoL and fatigue. In keeping with a previous study based on mindfulness interventions for chronic disease (Dantzer and Le Barbenchon, 2016), the present study shows a greater improvement in the psychological, emotional, and social dimensions of QoL than in the physical dimensions. The participants also reported a reduction in pain, and in both emotional and physical fatigue, which is a promising result for patients living with cancer. Unexpectedly, participants also reported an increase in nausea and vomiting. This result is probably explained by the stage of diseases of the included patients, most of whom were undergoing chemotherapy at the time of the study. Another possible interpretation is that, by learning mindfulness skills, patients increased their ability to identify their symptoms and pay more attention to their bodies. This ability could be useful to take care of the disease and increase self-care. A trend toward an improvement in anxiety and depression scores was observed, although this result did not reach statistical significance. Therefore, the assessment of quality of life scores seems more relevant to evaluate the impact of the MAEva program (McCloy et al., 2022). This study enabled us to calculate a suitable study sample size for future randomized controlled trials based on this outcome. Furthermore, each thematic session provides similar benefits, underlining the importance of all elements of the program.

The qualitative section shows that the patients learned skills using the ACT and mindfulness practices. More specifically, interviewed participants shared their satisfaction regarding the development of their ability to “let go”, as well as a close connection with an authentic self through their values, and their commitment to daily mindful practices. These new abilities could help them to deal with challenging emotions and thoughts that are unavoidable with a chronic disease like cancer (Pitman et al., 2018). Moreover, the MAEva program provided participants with a greater feeling of autonomy, competence, and connection with other persons living with the same disease. A recent study highlighted the influence of psychological needs, and more particularly the competence need, in predicting physical activity for patients with breast cancer during chemotherapy and recommended that health professionals should pay attention to the satisfaction of these needs (Fu et al., 2022). The qualitative results of the current study also highlight the overlap of ACT processes and their connection with the satisfaction of the three basic psychological needs described in the self-determination theory (Ryan et al., 2008). The participants' discourse illustrates that the MAEva program influenced the development of their intrinsic motivation and promoted their psychological flexibility.

This study extends existing knowledge regarding the potential of using meditation-based interventions for cancer patients in reducing distress and improving QoL. Indeed, this original program, with an open and circular design, was created very differently from the classic mindfulness programs delivered in a closed 8-week format. Engaging in formal meditation training, as offered in the MBSR program, is extremely difficult for cancer patients and sometimes inadvisable. They face significant stressors related to illness, treatment, and fatigue as well as multiple logistical obstacles (Zernicke et al., 2016; Peters et al., 2020). A few studies have highlighted the difficulties cancer patients face in meeting the demands of an 8-week program and the need to adapt interventions to make them less intensive (Eyles et al., 2015; Kubo et al., 2018). In the present study, the participants greatly appreciated the flexibility of the MAEva program (the possibility of missing sessions and making up for them) as well as the characteristics of the practices trained (short meditations [10–15 min], exercises proposing a systematic approach to regulating stress, informal practices, and theoretical contributions).

In addition, during this feasibility study, useful observations were made to assess the relevance of the MAEva program in the patients' real lives. Firstly, the recruitment did not encounter any difficulty, and the enrollment of 40 patients was much faster than expected (few refusals, patients motivated to participate). Secondly, an improvement in QoL was observed, with high levels of satisfaction and no negative consequences. Thirdly, the MAEva program is an intervention suitable for all cancer patients, at all stages of disease. Moreover, its implementation appeared simple and inexpensive to us, although we must consider that our center had psychology professionals trained in ACT and mindfulness available on site, and this may not be the case everywhere (Zhang et al., 2022). Lastly, the MAEva program is a written and structured intervention, and the responsibility for its delivery can be shared between different health professionals, facilitating implementation and maintenance within the hospital. However, it should be noted that the facilitators who took part in this study were all trained in each thematic session of the program. The satisfaction questionnaires completed by the healthcare professionals showed that they were motivated and firmly convinced of the utility of this program, which is an essential condition for the success of such a project (Hutchinson et al., 2021).

Our methods provide a practical strategy for conducting a larger, fully powered clinical trial, and we provide important insights for further work in the future. The question of the effectiveness of the MAEva program could be addressed via a randomized controlled trial, with quality of life as the main outcome for which we can calculate the study sample size. Qualitative analyses also suggest the importance of evaluating the flexibility processes of ACT. This could be done quantitatively in future larger studies using scales such as the Multidimensional Psychological Flexibility Inventory (MPFI) (Grégoire et al., 2020).

## Limitations

This study has some limitations. First, it was a single-arm feasibility study, and, thus, the lack of a control group and the small sample size make it impossible to draw firm conclusions regarding the potential efficacy of the intervention on participant-reported outcomes. The observed improvements in QoL and fatigue may simply represent natural improvements. In addition, the discrepancies between the number of men and women, between diagnoses and phases of the treatment, preclude any generalization of the results among cancer patients.

Second, the post-intervention interviews were conducted by a single facilitator, which may leave potential for social desirability bias. Indeed, it is possible that the patients questioned wanted to present themselves in a favorable light. However, the results of the satisfaction questionnaires and the post-intervention interviews concur to indicate that the participants appreciated the MAEva program. It helped them to cultivate presence, acceptance, commitment, and better regulation of stress, providing sufficient justification for further investigation of the effectiveness of this intervention through larger randomized clinical trials.

## Conclusion

This feasibility study provides encouraging evidence that an original, open, and circular program of ACT with mindfulness practices is acceptable for breast cancer patients (newly diagnosed or undergoing treatment). Although no causality could be definitively established from this study, our findings encourage the performance of additional randomized control trials, especially given the very low risks associated with the MAEva program. Future larger scale randomized studies involving diverse populations are necessary to establish the efficacy of the MAEva program. Pragmatic trials of this type of intervention within large healthcare systems will help document the effectiveness of implementing structured, low-cost programs that combine ACT and mindfulness practices, which are less demanding than conventional eight-week mindfulness programs, for cancer patients.

## Data availability statement

The raw data supporting the conclusions of this article will be made available by the authors, without undue reservation.

## Ethics statement

The studies involving humans were approved by the Ethics Committee or Comité de Protection des Personnes Ile de France X on 15 March 2021 under the number ID-RCB: 2021-A00601-40. The studies were conducted in accordance with the local legislation and institutional requirements. The participants provided their written informed consent to participate in this study.

## Author contributions

FB: Conceptualization, Formal analysis, Investigation, Methodology, Supervision, Validation, Visualization, Writing – original draft, Writing – review & editing. DB: Formal analysis, Project administration, Supervision, Validation, Visualization, Writing – original draft, Writing – review & editing. CH-S: Conceptualization, Methodology, Writing – review & editing. AS: Formal analysis, Writing – review & editing. LD: Investigation, Writing – review & editing. MG: Investigation, Writing – review & editing. VA: Investigation, Writing – review & editing. EF: Conceptualization, Formal analysis, Validation, Visualization, Writing – original draft, Writing – review & editing. AO: Conceptualization, Formal analysis, Methodology, Software, Writing – original draft, Writing – review & editing.
